# What fresh cell is this? Building a single-cell atlas of developing grass leaves in *Brachypodium distachyon*

**DOI:** 10.1093/plcell/koag206

**Published:** 2026-07-03

**Authors:** Róisín Fattorini

**Affiliations:** Assistant Features Editor, The Plant Cell, American Society of Plant Biologists, United States; Institute of Molecular Plant Sciences, Daniel Rutherford Building, Max Born Crescent, University of Edinburgh, Edinburgh EH9 3BF, United Kingdom

Poaceae is one of the largest plant families and contains grass species of ecological and agricultural importance. Grasses have distinct leaf morphologies with parallel venation, specialized stomatal complexes, and longitudinal leaf sheaths that encircle and protect the shoot apical meristem and immature leaves. Grass leaves are key biological structures that underpin form and function across many species. As such, understanding the mechanisms governing grass leaf development is important fundamentally as well as in applied contexts, such as crop improvement. Although there has been recent progress in characterizing leaf developmental processes, our understanding of how tissue development in grass leaves is coordinated remains limited. In maize, high-resolution transcriptomic techniques have helped to unravel aspects of these complex developmental mechanisms: single-cell RNA sequencing provided insight into cell-fate acquisition dynamics at the maize shoot apex (Satterlee et al. 2020), and single-nucleus RNA-sequencing has been used to identify key components of vein initiation in maize leaf primordia (Yi et al. 2026). Evidently, gene expression data with cellular resolution provided by single-cell transcriptomics is a powerful resource for disentangling grass leaf developmental mechanisms.

In their recent work, Lea Berg and colleagues (Berg et al. 2026) created a single-cell atlas of developing grass leaves in *Brachypodium distachyon*. *B. distachyon* is a tractable wild grass species with a leaf epidermis that consists of longitudinal cell files that form either bulliform cells only, or a mixture of specialized epidermal cells (eg stomata or hair cells) and pavement cells (Baird and Raissig 2026). The grass leaf blade is arranged into developmental zones where morphogenetic processes, including cell division and cell elongation, occur along a proximo-distal (base-to-tip) developmental gradient. Specific sections of developing leaf zones, thought to contain all leaf tissues at all developmental stages (Zhang et al. 2022; Nunes et al. 2023), were sampled along with the vegetative shoot apical meristem and early leaf primordia. These tissues were used to generate 9 datasets that were merged to produce a single-cell transcriptomic atlas consisting of 69,687 cells.

In a series of elegant experiments, the authors then used a priori knowledge of leaf epidermal development to further subset and investigate the *B. distachyon* epidermal lineage. Tissue and cell cycle information from the single-cell transcriptomic dataset was analyzed to identify putative marker genes for specific epidermal cell types based on gene expression. The expression patterns of these novel marker genes were then verified in planta using whole-mount RNA fluorescence in situ hybridization. A 2-component response regulator (*2cRR*), for example, was identified as a reliable marker gene for 2 stages of hair cell development (Fig. 1).

**Figure 1:**
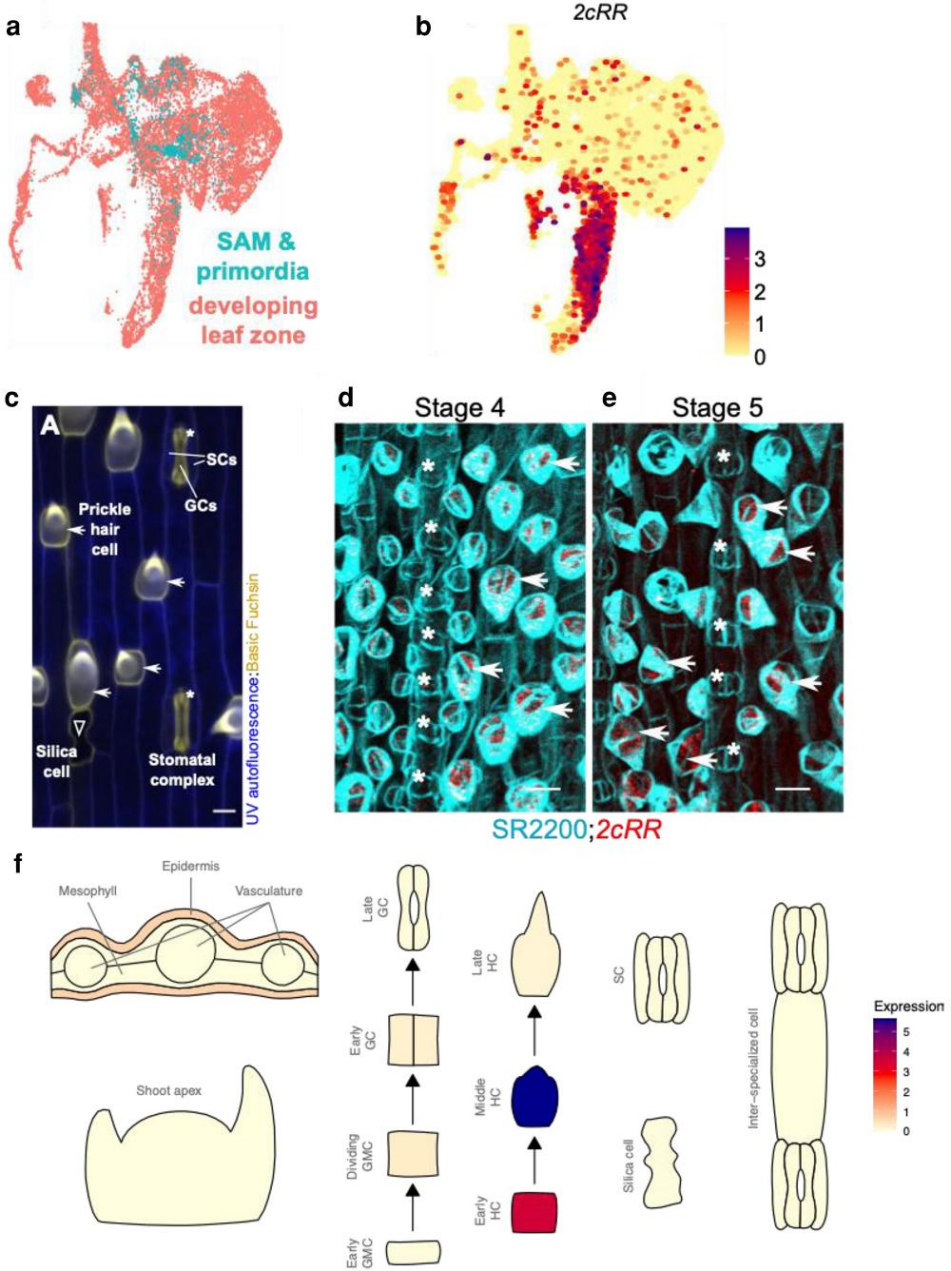
a) Epidermis UMAP plot showing the tissue type of 15,034 cells. b) Epidermis UMAP plot showing the expression levels of the 2 component response regulator *2cRR.* c) Confocal image of the *B. distachyon* mature abaxial leaf epidermis. Silica cells are labeled with triangles, stomatal complexes with asterisks, and prickle hair cells with arrows. d, e) Whole-mount RNA-FISH images of *2cRR* expression in the epidermis at stages 4 (d) and 5 (e). f) Gene expression visualization for *2cRR* (BdiBd21-3.3G0655900) across tissue types using the *Brachypodium* leaf single cell atlas online gene expression viewer (https://shiny.ips.unibe.ch/). A to e were taken from Figure 2 of Berg et al. (2026).

Gene regulatory network analyses were conducted in the stomatal lineage cluster. These analyses used the single-cell transcriptomic dataset to build networks that incorporated transcription factor motif information. The regulons produced consisted of a transcription factor and its potential downstream regulatory targets (targetome) in a given cell type (Ferrari et al. 2022). This analysis identified several transcription factors already known to be involved in leaf and stomatal development, demonstrating the potential of this method to identify novel regulators at key developmental stages.

Among the top regulons found were for the bHLH transcription factors *BdMUTE* and *BdFAMA* in the dividing guard mother cell (GMC) and early guard cell (GC) clusters, respectively. While both transcription factors are expressed in GMCs and recognize identical binding motifs, they have distinct functions in regulating stomatal development. Specifically, *BdMUTE* regulates the orientation of GMC cell division (Spiegelhalder et al. 2024), while *BdFAMA* regulates GC pore formation and differentiation (McKown et al. 2023). This difference in function was reflected in the transcription factor targetomes, with target genes exclusively or preferentially (based on the coexpression strength between the transcription factor and target gene pair) associated with *BdMUTE* tending to be involved in cell division and those associated with *BdFAMA* more likely to be involved in morphogenesis and cell wall biology. The consistency between known transcription factor function and the likely functional roles of unique or preferential target genes demonstrates that the single-cell transcriptomic atlas can be used to identify distinct regulatory gene networks when gene expression of the transcription factor occurs within the cell type of interest. In this case, the precision of these inferences was enough to identify functional divergence between 2 coexpressed bHLH transcription factors. In the future, this method could be used for comparative analyses with other single-cell transcriptomic datasets, comparing the targetomes of the same transcription factors in different species or biological contexts.

This study provides a spatially and pseudotemporally resolved single-cell transcriptomic atlas of the *B. distachyon* vegetative shoot apical meristem and developing leaves. Proof-of-concept experiments demonstrated that this dataset is useful for identifying novel marker genes, heterogenous cell types, and candidate genes for downstream functional analyses. This atlas is a valuable resource for grass research and the broader plant science community accessible through an online gene expression viewer (https://shiny.ips.unibe.ch/).

## Recent related articles in *The Plant Cell*

Yi et al. (2026) created a single-nucleus transcriptomic atlas of maize primordia and investigated the patterning mechanisms underlying vein development in leaf primordia of maize and rice.

Wu et al. (2024) investigated the regulation of stomatal development in the *Arabidopsis thaliana* leaf epidermis. They found that the activation or suppression of MPK3/MPK6, which are components of the mitogen-activated protein kinase cascade, in mesophyll cells or the epidermis is sufficient to change the differentiation of stomata and further investigated the molecular mechanisms underlying this phenotype.

## Data Availability

No new data were generated or analysed in support of this research.
